# Recurrent uveitic macular edema managed with intravitreal faricimab injection

**DOI:** 10.1186/s12348-025-00478-6

**Published:** 2025-03-13

**Authors:** Negin Yavari, Dalia El Feky, Frances A. Anover, Khiem Nguyen, Azadeh Mobasserian, Quan Dong Nguyen, Christopher Or

**Affiliations:** Spencer Center for Vision Research, Byers Eye Institute at Stanford University, 2452 Watson Court, Suite 200, 94303 Palo Alto, CA USA

**Keywords:** Retinal vasculitis, Uveitic macular edema, Intravitreal anti-vascular endothelial growth factor, Intravitreal faricimab injection, Birdshot chorioretinopathy

## Abstract

**Purpose:**

To present a case of recurrent uveitic macular edema (UME) treated with intravitreal faricimab injection.

**Methods:**

Single case report from a tertiary referral center.

**Observations:**

A 63-year-old Caucasian female presenting with recurrent UME due to birdshot chorioretinopathy (BSCR) in both eyes. UME had been treated with multiple agents including intravenous methylprednisone, posterior subtenons and intravitreal triamcinolone acetonide injection, dexamethasone implant, mycophenolate mofetil, and adalimumab with limited improvement and development of intolerance. Moreover, optical coherence tomography also revealed recurrence of UME with subretinal fluid in both eyes. After treatment with one dose of intravitreal faricimab injection, complete resolution of UME was achieved and maintained for three months.

**Conclusion:**

The findings of this case hint towards the potential simultaneous effect of angiopoietin-2 blockade along with vascular endothelia growth factor A inhibition by faricimab in managing treatment-resistant UME. Nonetheless, more studies focusing on the role of intravitreal faricimab in UME are required.

## Introduction

Uveitic macular edema (UME) is a frequent complication in uveitis patients and is a leading cause of significant visual impairment. In the Multicenter Uveitis Steroid Treatment (MUST) Trial, macular edema was present at enrollment in 40% of eyes with uveitis with a similar frequency for patients with intermediate uveitis, posterior uveitis, and panuveitis [1–3].

Despite the numerous available drugs for its treatment, at least a third of patients with UME fail to achieve satisfactory improvement in visual acuity [2]. For decades, the first-line treatment of noninfectious uveitis and macular edema have been corticosteroids administered orally, topically, or through either a periocular or intravitreal injection [2, 4]. In the MUST Trial, 62% of eyes with UME treated with systemic medications required regional corticosteroid injections for macular edema [3]. Moreover, it has been shown that intravitreal injection of corticosteroids is superior to other routes of injections [5]. However, risk of developing side effects including cataract, glaucoma, or drug intolerance require the addition of other groups of therapeutic drugs [5–7]. Alternative treatments that could be considered are oral corticosteroids, steroid-sparing immunosuppressives, anti-vascular endothelial growth factor (VEGF) injections, and biologic medications [6]. However, the Macular Edema Ranibizumab versus Intravitreal anti-inflammatory Therapy (MERIT) trial demonstrated that VEGF inhibition with ranibizumab was less effective than the dexamethasone implant in the treatment of UME [8]. Furthermore, many of these medications are used off-label for UME, and there is limited data on their effectiveness in reducing the edema [2, 6].

Faricimab is the first intravitreal therapy designed to simultaneously target VEGF-A and angiopoietin-2 (Ang-2) through a bispecific monoclonal antibody [9, 10]. This approach aims to stabilize retinal blood vessels and inhibit the VEGF drive [9, 10]. Faricimab has recently been approved for the treatment of diabetic macular edema (DME), neovascular age-related macular degeneration (nAMD), and macular edema associated with retinal vein occlusion [11]. However, its use and potential effects on UME are unknown.

We report a case of a patient with birdshot chorioretinopathy (BSCR) with retinal vasculitis, and persistent UME. The patient had multiple intolerances to other therapeutic options. Persistent UME was eventually treated with intravitreal faricimab, resulting in significant improvement in macular edema.

## Case presentation

A 63-year-old Caucasian female came to the Uveitis Clinic at a tertiary eye center for the management of BSCR complicated by retinal vasculitis and UME. Other ocular history included laser-assisted in situ keratomileusis and posterior subtenons triamcinolone acetonide (PS-TAAC) injection once in both eyes with incomplete response. Her medical history includes history of pancreatitis. Upon examination, best corrected visual acuity (BCVA) was 20/60 in the right eye (OD) and 20/200 in left eye (OS). Intraocular pressure (IOP) was 13 and 14 in OD and OS, respectively. A full laboratory work was performed; all came back non-revealing except for human leukocyte antigen (HLA)-A29 positivity. Anterior segment examination showed no inflammation in the anterior chamber; however, the vitreous revealed 1 + haze in both eyes. Fundus examination revealed clinically significant macular edema in both eyes. Wide angle fluorescein angiography demonstrated diffuse leakage from macula, optic disc and retinal vessels with peripheral non-perfusion in both eyes. Moreover, optical coherence tomography (OCT) showed disrupted foveal contour with significant UME and subretinal fluid in both eyes.

**Fig. 1:**
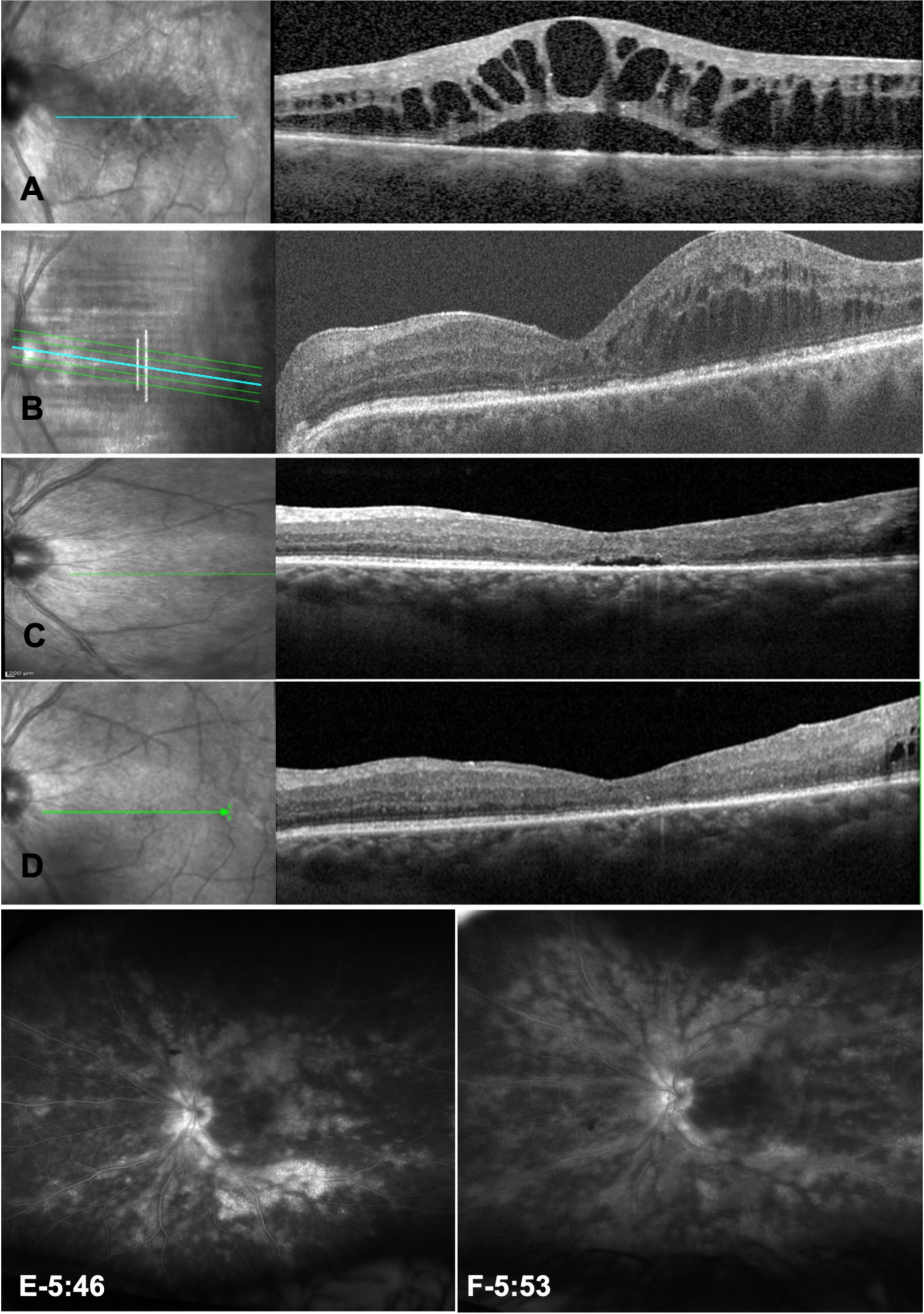
Posterior imaging of the left eye at presentation and after faricimab injection. **A)** Optical coherent tomography revealed cystoid macular edema and subretinal fluid at presentation. **B)** Significant cystoid macular edema prior intravitreal faricimab injection. **C)** Mild cystoid macular edema one month after intravitreal faricimab injection. **D)** Resolution of cystoid macular edema three months after intravitreal faricimab injection. **E)** Diffuse leakage from the optic nerve, peripheral retinal vasculature, and macular edema at presentation. **F)** Decreased leakage within the macula and optic nerve with persistent leakage from the retinal vasculature after faricimab injection

The patient subsequently received three cycles of monthly intravenous methylprednisone infusions. Despite such treatment, macular and perivascular leakage persisted, leading to the initiation of adalimumab therapy. However, the patient experienced skin reactions at the injection sites and discontinued adalimumab, switching to mycophenolate mofetil 1500 mg twice daily. Since UME remained significant in both eyes, a suprachoroidal injection of TAAC was performed which improved the UME for three months, but the UME recurred (Fig. 1A) despite repeated suprachoroidal TAAC injections [12].

Given the previous incomplete response to other therapies and patient’s refusal of intravitreal steroids due to potential side effects, an intravitreal faricimab was injected in the OS. After three months of follow up, the injection was well tolerated, and UME showed significant resolution in OCT (Fig. 1B-D) with BCVA of 20/60 in OD and 20/50 in OS, despite continued activity of BSCR demonstrated on fluorescein angiogram (Figure E-F). Within the next six months, UME recurred, and appropriate treatment was administered.

## Discussion

Macular edema is the leading cause of vision loss in patients with uveitis [2, 13, 14]. Managing UME when there is no active uveitis presents significant difficulties and often requires exploring new treatment options [13, 14]. In this case, we presented a case of a 63-year-old Caucasian female with BSCR, retinal vasculitis, and UME, who received an intravitreal faricimab injection. Administration of intravitreal faricimab was associated with resolution of recurrent UME after one dose, and by 3 months, the patient maintained the benefit.

To date, there is no widely accepted treatment for managing UME. Traditionally, anti-inflammatory therapy such as corticosteroids is the primary approach for managing UME [14]. Corticosteroids administered orally, intravitreally, or via subtenon injection are used to treat UME due to their potential systemic anti-inflammatory effects, but their long-term use is associated with significant side effects. In cases where oral corticosteroids are not sufficiently effective or produce intolerable side effects, corticosteroid treatments can be delivered through periocular and/or intravitreal methods [13, 14]. Intravitreal corticosteroid injections, such as dexamethasone implants, have shown effectiveness in reducing macular thickness and improving visual acuity in patients with UME. The MERIT trial also demonstrated that dexamethasone implant was significantly better at treating persistent or recurrent UME compared to intravitreal ranibizumab or intravitreal methotrexate [8]. Another option would be the use of suprachoroidal triamcinolone acetonide which also showed improvement in visual acuity and macular edema [12]. Despite various treatment options, there are still patients with recalcitrant disease and recurrent macular edema necessitating repeat therapies and injections [13, 14].

Faricimab, a biphasic antibody targeting both VEGF-A and Ang-2, a novel agent for the treatment of DME, neovascular AMD and retinal vascular diseases, has demonstrated an overall favorable efficacy in randomized controlled trials [9, 10, 15]. No specific anti-VEGF treatment regimen for UME has been proposed in cases where the use of immunosuppressive therapies is limited. The MERIT trial evaluated ranibizumab as a treatment option for UME. Even though it showed efficacy in reducing UME, its efficacy was inferior to that of the dexamethasone implant [8]. By targeting both VEGF-A and Ang-2, faricimab may offer a broader approach to the treatment of retinal diseases, potentially making it more effective than ranibizumab, which only targets VEGF-A [10, 16].

It is well known that a major factor leading to the development of UME is the breakdown of blood-retinal barrier due to chronic inflammation, which involves multiple pathways [14], including inflammation-induced vascular instability, leakage and degradation of tight junction proteins by intracellular phosphorylation, which are partially mediated by VEGF [17]. Ang-2 also contributes to this process by destabilizing vascular endothelium and promoting inflammation [18]. In a study by Oshima et al., Ang-2 was shown to enhance VEGF sensitivity in retinal vessels, leading to extensive neovascularization. They suggested that blocking Ang-2 expression with VEGF blockade could be a potential treatment for retinal neovascularization [19]. Therefore, faricimab, not only inhibit vascular leakage by targeting VEGF-A, but also increases vascular stability by reducing inflammation through Ang-2 blockade [10, 11]. Moreover, the simultaneous inhibition of both VEGF-A and Ang-2 may lead to a potentially longer-lasting effect [20, 21].

Faricimab may be particularly beneficial in specific situations in the management of UME. Patients who require long term local therapy due to either recalcitrant macular edema or intolerance to other forms of therapy may benefit from extended dosing regimen using faricimab. The burden of multiple treatments for UME is multifaceted, involving significant economic costs, clinical challenges, and impacts on patients’ quality of life [2]. The potential extended durability of faricimab, as demonstrated in previous trials for AMD and DME, may be helpful in the management of UME and remains to be investigated [22, 23]. Moreover, faricimab can be considered as a therapy for patients who are at risk of developing steroid-related side effects including glaucoma or cataract [24, 25]. Since it does not cause increased intraocular pressure or accelerate cataract formation, faricimab can also be a better local therapy option for younger patients to avoid long-term complications of corticosteroid use [21]. However, it should be stated that faricimab may only treat the macular edema and the management of the underlying uveitis should still be emphasized.

The limitation of our report is that it is based on a single patient, which limits the generalizability of its findings. Without a comparison to other treatment options, it is difficult to assess the efficacy of faricimab when compared to existing treatments for UME. Therefore, further studies are required to clarify and validate the use of faricimab in the therapeutic regimen for the management of UME.

## Conclusion

The index case report demonstrates the potential efficacy of intravitreal faricimab in the management of recalcitrant UME. However, further studies are required to elucidate its role in the management of UME.

## Data Availability

No datasets were generated or analysed during the current study.

## Abbreviations

ANG-2: Angiopoietin-2
CDVA: Corrected distance visual acuity
DME: Diabetic macular edema
HLA: Human leukocyte antigen
IOP: Intraocular pressure
MERIT: Macular Edema Ranibizumab versus Intravitreal anti-inflammatory Therapy
MUST: Multicenter Uveitis Steroid Treatment
nAMD: Neovascular age macular degeneration
OCT: Optical coherence tomography
PS-TAAC: Posterior subtenons triamcinolone acetonide
UME: Uveitic macular edema
VEGF: Vascular endothelial growth factor

## References

[1] Kawali A et al (2022) Intensive topical interferon therapy in uveitic macular edema. Indian J Ophthalmol 70(8):2986–2989 35918958 10.4103/ijo.IJO_3210_21 PMC9672726

[2] Teper SJ (2021) Update on the management of uveitic macular edema. J Clin Med 10(18):4133 34575244 10.3390/jcm10184133 PMC8470573

[3] Group MUSTTR (2010) The multicenter uveitis steroid treatment trial: rationale, design, and baseline characteristics. Am J Ophthalmol 149(4):550–561e10 20097325 10.1016/j.ajo.2009.11.019 PMC2975449

[4] Jabbour M et al (2024) Efficacity and safety of the Fluocinolone acetonide implant in uveitic macular edema: A Real-Life study from the French uveitis network. J Personalized Med 14(3):245 10.3390/jpm14030245 PMC10971732 38540987

[5] Thorne JE et al (2019) Periocular triamcinolone vs. intravitreal triamcinolone vs. intravitreal dexamethasone implant for the treatment of uveitic macular edema: the periocular vs. INTravitreal corticosteroids for uveitic macular edema (POINT) trial. Ophthalmology 126(2):283–295 30269924 10.1016/j.ophtha.2018.08.021 PMC6348060

[6] Deuter CM et al (2017) Tocilizumab in uveitic macular edema refractory to previous Immunomodulatory treatment. Ocul Immunol Inflamm 25(2):215–220 26731514 10.3109/09273948.2015.1099680

[7] Lowder C et al (2011) Dexamethasone intravitreal implant for noninfectious intermediate or posterior uveitis. Arch Ophthalmol 129(5):545–553 21220619 10.1001/archophthalmol.2010.339

[8] Acharya NR et al (2023) Intravitreal therapy for uveitic macular Edema—Ranibizumab versus methotrexate versus the dexamethasone implant: the MERIT trial results. Ophthalmology 130(9):914–923 37318415 10.1016/j.ophtha.2023.04.011 PMC10524707

[9] Heier JS et al (2022) Efficacy, durability, and safety of intravitreal faricimab up to every 16 weeks for neovascular age-related macular degeneration (TENAYA and LUCERNE): two randomised, double-masked, phase 3, non-inferiority trials. Lancet 399(10326):729–740 35085502 10.1016/S0140-6736(22)00010-1

[10] Khanani AM et al (2023) The real-world efficacy and safety of faricimab in neovascular age-related macular degeneration: the TRUCKEE study–6 month results. Eye 37(17):3574–3581 37173428 10.1038/s41433-023-02553-5 PMC10686385

[11] Shirley M (2022) Faricimab: first approval. Drugs 82(7):825–830 35474059 10.1007/s40265-022-01713-3

[12] Thomas J et al (2022) Triamcinolone acetonide injectable suspension for suprachoroidal use in the treatment of macular edema associated with uveitis. Expert Rev Ophthalmol 17(3):165–173 36060305 10.1080/17469899.2022.2114456 PMC9438525

[13] Foster CS, Vitale AT (2013) Diagnosis & treatment of uveitis. JP Medical Ltd.

[14] Preble JM, Foster CS (2015) Uveitic macular edema: A stepladder treatment paradigm.

[15] Sahni J et al (2019) Simultaneous Inhibition of angiopoietin-2 and vascular endothelial growth factor-A with faricimab in diabetic macular edema: BOULEVARD phase 2 randomized trial. Ophthalmology 126(8):1155–1170 30905643 10.1016/j.ophtha.2019.03.023

[16] Rosenfeld PJ et al (2006) Ranibizumab for neovascular age-related macular degeneration. N Engl J Med 355(14):1419–1431 17021318 10.1056/NEJMoa054481

[17] Lin D et al (2023) Synergistic effect of combined Sub-Tenon triamcinolone and intravitreal Anti-VEGF therapy for uveitic macular edema. Drug Design, Development and Therapy, pp 1055–1066 10.2147/DDDT.S353251 PMC9004729 35422612

[18] Suzuki K et al (2023) Involvement of angiopoietin 2 and vascular endothelial growth factor in uveitis. PLoS ONE 18(11):e0294745 38015876 10.1371/journal.pone.0294745 PMC10683998

[19] Oshima Y et al (2004) Angiopoietin-2 enhances retinal vessel sensitivity to vascular endothelial growth factor. J Cell Physiol 199(3):412–417 15095288 10.1002/jcp.10442

[20] Sharma A et al (2020) Faricimab: expanding horizon beyond VEGF. Eye 34(5):802–804 31695160 10.1038/s41433-019-0670-1 PMC7182558

[21] TENAYA, Investigators L (2024) TENAYA and LUCERNE: Two-Year results from the phase 3 neovascular Age-Related macular degeneration trials of faricimab with Treat-and-Extend dosing in year 2. Ophthalmology 10.1016/j.ophtha.2024.02.014 38382813

[22] Khanani AM et al (2024) TENAYA and LUCERNE: Two-Year results from the phase 3 neovascular Age-Related macular degeneration trials of faricimab with Treat-and-Extend dosing in year 2. Ophthalmology 10.1016/j.ophtha.2024.02.014 38382813

[23] Eter N et al (2022) YOSEMITE and RHINE: phase 3 randomized clinical trials of faricimab for diabetic macular edema: study design and rationale. Ophthalmol Sci 2(1):100111 36246184 10.1016/j.xops.2021.100111 PMC9559760

[24] Bojikian KD et al (2021) Incidence of and risk factors for steroid response after cataract surgery in patients with and without glaucoma. J Glaucoma 30(4):e159–e163 33428351 10.1097/IJG.0000000000001785

[25] Roberti G et al (2020) Steroid-induced glaucoma: epidemiology, pathophysiology, and clinical management. Surv Ophthalmol 65(4):458–472 32057761 10.1016/j.survophthal.2020.01.002

